# Medical students developing confidence and patient centredness in diverse clinical settings: a longitudinal survey study

**DOI:** 10.1186/s12909-016-0689-y

**Published:** 2016-07-15

**Authors:** Ruth McNair, Leonie Griffiths, Katharine Reid, Hannah Sloan

**Affiliations:** The Department of General Practice, The University of Melbourne, 200 Berkeley St, Carlton, 3053 VIC Australia; Northern Clinical School, Melbourne Medical School, the University of Melbourne, 185 Cooper St, Epping, 3076 Australia; Melbourne Medical School, The University of Melbourne, Parkville, VIC 3010 Australia

**Keywords:** Clinical education, Community based education, Qualities/skills/values/attitudes

## Abstract

**Background:**

Medical student clinical confidence and positive attitudes to patient centredness are important outcomes of medical education. The clinical placement setting is regarded as a critical support to these outcomes, so understanding how the setting is influential is important. The aim of this study was to compare students’ attitudes towards patient-centredness and clinical confidence as they progressed through their medical course, and understand the influence of diverse clinical placement zones.

**Methods:**

Students at one Australian medical school completed a questionnaire at the beginning of second year and at the end of their third year of medical training. The questionnaire measured attitudes to patient centred care, clinical confidence, role modelling experiences and clinical learning experiences. Descriptive analyses investigated change in these attitudes over time. Repeated measures analysis of variance was used to assess the influence of placement location on each variable of interest. Responses to two open-ended questions were also coded by two researchers and themes were identified.

**Results:**

Student confidence increased over the course of two years of clinical training (*p* < 0.001), but attitudes to patient centredness (*p* = 0.933) did not change. The location of clinical placements (urban, outer urban and rural) was unrelated to levels of confidence or patient centredness. Students had positive attitudes towards patient-centredness throughout, and noted its importance in contributing to quality care. Patient-centred care was encouraged within the clinical placements, and was influenced by positive and negative role modelling, direct teaching, and opportunities to practise patient-centred care.

**Conclusions:**

A new generation of doctors with a strong patient-centred focus is emerging. Medical schools have a responsibility to facilitate clinical placements that will support the acquisition and maintenance of skills in patient centred care through positive role modelling.

## Background

Clinical confidence and patient centredness are highly prized outcomes of medical education amongst educators and consumers alike [1, 2]. These characteristics result in a capable and humane approach to holistic patient care. Further, a medical school that encourages them is partly satisfying recent calls for social accountability [3]. Clinical confidence is the self-perceived ability to deal with clinical scenarios, and while it does not necessarily correlate with competency, it is nevertheless a pre-requisite for students to be able to fully participate in clinical activities [4]. Patient centredness is a set of attitudes or value system that counteracts the limitations of the conventional biomedical model by bringing the patient agenda into focus. Mead and Bower have provided a theoretical framework that defines patient centredness using five dimensions: a biopsychosocial perspective, patient-as-person (understanding the patient’s experience of their illness), shared power (therefore increasing patient involvement), therapeutic alliance, and doctor-as-person (self-awareness of their own subjectivity) [5].

Patient centredness is found to deteriorate as students progress through their medical studies [6]. Positive attitudes to patient centredness are often acquired through direct teaching, as well as via clinical experience and appropriate role modelling, often termed the ‘hidden curriculum’ [7]. The hidden curriculum may also have a negative impact on both confidence and patient-centredness. The influences of socialisation into the culture of medicine can be subtle but powerful and include sources such as the type of clinical setting, and the nature of both patient interactions and clinical mentoring [8]. Haidet (2010) suggests that patient centredness “challenges prevailing professional norms”, and that non-patient centred behaviour by clinicians is the most important influence on student behaviour [9].

One element of the hidden curriculum is the style of clinical placement. For example, traditional hospital-based placements with multiple short rotations through various disciplines (block learning) do not allow students to participate in longer term care of patients, and also involve frequent changes in clinical supervisor [10, 11]. Traditional placements have been criticised as difficult learning environments; preventing students from forming effective therapeutic relationships with patients [12] and minimising their role in the health care team [10]. In this environment, medical student attitudes towards patients have been found to shift from being patient-centred to doctor-centred and disease focused (more so for male students) [13], due to an emphasis on biomedicine rather than patient perspectives [14].

Community-Based Medical Education (CBME) and Longitudinal Integrated Clerkships (LICs) are seen as two possible alternatives to traditional placements. Both approaches support longer-term contact with the same patients and clinical supervisors, therefore allowing students to build relationships, understand continuity of care, have meaningful mentoring and take some responsibility for patient care [15, 16]. These experiences all contribute to improved clinical confidence in a number of areas [17, 18]. They also facilitate the development of patient centredness through the use of a more integrated and patient-orientated approach to learning [16, 19]. CBME within rural settings is found to have further positive impacts on student patient-centredness [15]. CBME is increasingly popular, with participating medical students demonstrating higher levels of humanism and patient centredness at graduation [20].

For the current study, our medical school was uniquely placed to assess the impact of diverse clinical placement settings on attitudes including confidence and patient centredness for students commencing a new medical course in 2011. The course is a post-graduate entry, four-year Doctor of Medicine (MD) degree course. The first year is University based, years two and three comprise clinical training with summative assessment, and the fourth year includes a research project and six months of capstone clinical training with formative assessment. The 330 students in the first cohort of the MD were allocated to one of three different clinical placement zones for their second and third years (inner metropolitan, outer metropolitan or rural). Students were allocated to a clinical zone on the basis of personal preference and their selection ranking and were then randomly allocated to specific clinical schools within zones. Medical school monitoring of the academic achievement of students within each zone showed no systematic differences in average achievement in the course.

The site of clinical placements varied for the three zones. Inner metropolitan students were based almost entirely in tertiary teaching hospitals using block placements. Outer metropolitan students spent four days a week in a major teaching hospital and attended the same general practice for one day per week for two years (the Primary Care Community Base [PCCB] program). Rural students were based in regional hospitals with some attending general practice for two days per week (the Extended Rural Cohort [ERC]). The aims of the PCCB and ERC programs were to enable students to become competent, patient-centred, humanistic, culturally competent, adaptable and well equipped to serve in any community.

### Study purpose and research questions

The purpose of this study was to compare students’ attitudes to patient centredness and their own clinical confidence as they progressed through the medical course, and to identify any differences according to their clinical placement location. We anticipated that over time, student clinical confidence would increase. We predicted that attitudes to patient centredness would become more negative overall, which is commonly seen in the literature related to a lack of student self-efficacy and lack of patient-centred role models [6]. However, we predicted that attitudes to patient centredness would be enhanced in the outer metropolitan and rural zones, because there would be more longitudinal connection with community-based clinicians as role models and with specific patients in general practice. This hypothesis was based in the fact that several of the dimensions of the patient centredness framework are more commonly practiced in community care particularly biopsychosocial perspectives and patient-as-person. The primary question was ‘how did students’ attitudes to patient centredness and their self-perceived clinical confidence change as they transitioned through two clinical years of the medical course? We posed two secondary questions: ‘Did the location of the clinical experiences impact on attitudes to patient centredness and self-perceived confidence?’ and ‘What influence did role modelling have on patient centredness and confidence?’ Results pertaining to each of these questions are reported in this article.

## Methods

### Study design

This longitudinal, survey-research design, comprised a survey administered twice over two years to the same cohort of medical students in an Australian medical school. The survey was created following a literature review on medical student experiences in clinical training, patient centredness and CBME (Table 1). Most items within the survey were adapted from previously validated measures including the Patient-Practitioner Orientation Scale [21], the Harvard medical school Cambridge Integrated Clerkship evaluation [16], and the C3 instrument – Communication, Curriculum and Culture [7].

**Table 1:** Survey items in five domains

Domain	Items	5-point Likert Scales
Patient Centred Care	1. Patients should be treated as if they were partners with the doctor, equal in power and status 2. When doctors ask a lot of questions about a patient’s background, they are prying too much into personal matters 3. How good doctors are at diagnosis and treatment is equally as important as how they relate to patients 4. When discussing a patient’s case history with another doctor, it is appropriate to refer to the patient as their diagnosis 5. A treatment plan cannot succeed if it is in conflict with a patient’s lifestyle or values 6. Being involved in seeing the same patients over long periods of time, improves patient care 7. The doctor has an important role in advocating for patient needs 8. The doctor is the one who should decide what is discussed during a visit 9. Doctors should play a greater role in disease management than in illness prevention	1 = Strongly Disagree to 5 = Strongly Agree
Confidence	1. Dealing with ethical dilemmas 2. Seeing patients independently 3. Relating to a diverse patient population 4. Being a self-reflective practitioner 5. Explaining medical information to patients 6. Contributing to patient care 7. Describing the natural history of illness over time	1 = Very Unconfident to 5 = Very Confident
Role Modelling	1. I often observed my supervisors communicate concern and interest in patients as unique persons 2. How I have been taught to treat patients is different to what I witnessed in my clinical experiences 3. My supervisors were more concerned with developing good rapport with patients, than meeting strict time deadlines for patient consultations 4. I often observed my supervisors encourage patients’ participation in their own care 5. I was often provided with feedback on how well I communicated with or listened to the patient and their emotional needs 6. My supervisors encouraged me when I made an effort to get to know patients as unique people	1 = Strongly Disagree to 5 = Strongly Agree
Clinical Learning Experience	1. My clinical supervisors were skilled teachers 2. I had a clear idea what was expected of me in the clinical environment 3. I received clear feedback from my clinical supervisors 4. My clinical supervisors were skilled clinicians 5. There was regular protected time for teaching in the clinical settings 6. There was no attempt to plan for my individual learning needs in the clinical environment 7. I had ample opportunity to see patients independently 8. I had the opportunity to follow up patients over time 9. I would prefer to have more community-based clinical experience	1 = Strongly Disagree to 5 = Strongly Agree

The survey was piloted with 19 medical students (9 male and 10 female) from the final cohort of the previous medical course who were in their third year of clinical training. The survey was also reviewed by medical education experts. Minor adjustments to improve clarity were then made to the survey.

Ratings of role modelling and clinical learning experiences were only included in the final survey, as students had not had exposure to the clinical environment prior to the first survey.

Two open-ended questions were also included in the final survey:

1. *Do you feel your clinical experiences encouraged you to practice patient-centred care?*
  Students could check yes or no, then provide an explanation
2. *What were the most positive and negative examples of role modelling of professional behaviour that you witnessed during your clinical experiences?*

Participants were provided with information about the study and invited to complete the paper-based survey at the beginning or conclusion of lectures or tutorials held in their clinical location. The research information and an invitation to participate was delivered by a member of the research team not involved with the medical course, to reduce any perceived pressure to participate in the study. Students completed the initial survey during the first month of their clinical training (March 2012), and the final survey at the end of their two years of clinical training (October to November 2013). Student responses were linked over time using their unique student identifier. The research received approval from the University of Melbourne Human Research Ethics Committee (approval no: 1137261). Student consent to participate was implied by the voluntary completion of the surveys.

### Data treatment

The four domains of the survey were examined using Cronbach’s alpha to determine the degree to which each formed an internally consistent scale. Cronbach’s alpha provides a measure of whether items comprising a scale are measuring the same construct and was used because the scales used were drawn from pre-existing instruments. Items were removed from each scale which led to appreciable increases in the Cronbach’s alpha value within our study, resulting in internal consistencies at the two time points for patient centred care of 0.62 and 0.62, and for students’ clinical confidence of 0.75 and 0.80. Cronbach’s alpha for the role modelling and clinical learning experiences scale at time two were 0.72 and 0.76 respectively. Combined measures of patient centred care (9 items), clinical confidence (7 items), role modelling (6 items) and clinical learning experiences (9 items) were constructed by averaging the scores for the items comprising the scale.

### Data analysis

The quantitative analyses had two aims. First, we analysed differences in students’ self-perceived patient centredness and their confidence in their skills as a clinician from the beginning to the end of two years of clinical training. Second, we examined variations across clinical settings. For these two steps, we undertook a repeated measures analysis of variance with measurement occasion and the three aspects of patient care as within subjects factors and clinical setting as a between subjects factor.

The qualitative analysis comprised a thematic analysis of the written answers to the two open-ended questions in the final survey. Two researchers independently coded comments under emerging themes. They then compared and discussed the themes they had arrived at independently and noted their similarities and resolved discrepancies. Analyses focused on perceptions of patient centredness, whether role modelling in different clinical settings appeared to be influential from students’ perspectives, and the direction of influence. Key themes arising were also compared and contrasted for each of the three placement zones.

## Results

### Participants

All 330 second year medical students from The University of Melbourne were invited to participate in the study. Two hundred and seventy-three students (83%) voluntarily completed the first survey in the beginning weeks of their placements at nine different clinical sites. A total of 275 students (83%) completed the final survey in the final weeks of their placements at the end of their second year of clinical training. In total, 203 matched questionnaires were available for analysis (62% of the cohort). Of the 203 participants, 56% were female, and 93% were domestic rather than international students. Students undertook their clinical training in hospital settings in inner metropolitan (46%), outer metropolitan (30%) and rural (24%) clinical zones. Most of these students identified culturally as Australian (51%), or as Australian and another cultural group (34%), with smaller proportions of students identifying as Asian (10%), or another cultural group (5%). We did initial comparisons between students who only completed the first survey and those who completed both time points. These preliminary analyses revealed no statistical differences between students who commenced the study and did not complete both time points, compared to those who remained in the study. The demographics for the matched sample were similar to the entire cohort (52% female students, 93% domestic students and 52%, 28% and 20% allocated to inner metropolitan, outer metropolitan and rural clinical zones). Written responses were provided to the two open-ended questions by 99 (49%) and 120 (59%) participants respectively.

### Change over time in patient centredness and confidence in clinical skills

Overall, student ratings increased from the beginning to the end of two years of clinical training; however, the degree to which this occurred depended on the domain, F (2, 396) = 39.57, *p* < 0.001, η_p_^2^ = 0.17. Pairwise comparisons suggested that, on average, there was growth in student confidence over the course of two years of clinical training (*p* < 0.001), but that average ratings for attitudes to patient centredness (*p* = 0.933) did not vary from the beginning to the end of the two years of clinical training (Table 2). There was no statistically significant variation between clinical zones on any of the measures, F (4, 396) = 2.39, *p* = 0.051.

**Table 2:** Means (and Standard Deviations) of Students’ Clinical Confidence and Patient Centredness at the Beginning and the End of Two Years of Clinical Training

Domain	Inner metropolitan (*n* = 93)		Outer metropolitan (*n* = 61)		Rural (*n* = 48)	
	Time 1	Time 2	Time 1	Time 2	Time 1	Time 2
Clinical confidence	3.6 (.42)	3.9 (.40)	3.6 (.57)	4.0 (.47)	3.6 (.51)	4.0 (.43)
Patient centredness	4.0 (.39)	4.0 (.35)	4.0 (.36)	4.0 (.41)	4.0 (.35)	3.9 (.35)

### Influences on patient centredness

The responses to the open-ended question on patient centredness provided a more in-depth understanding of student perceptions of patient centredness and their perceptions of key influences. These responses demonstrated that patient-centred care (PCC) was highly regarded. All but seven students (97%) agreed that the clinical experience encouraged them to practice patient-centred care and students from all clinical zones expressed similar attitudes. Several students chose to define PCC in their answer, and these definitions fitted well with the dimensions described by Mead and Bower (2000), including seeing the patient as a whole, understanding the patient perspective, creating a shared agenda, and tailoring management to the individual. For example,

“You learn that a medical intervention is rarely all that a person needs. Mental, social, financial, emotional support are frequently required.” (#13, male, outer metropolitan)

Several students described what had encouraged them to practice PCC, including seeing improved patient outcomes (16 responses), helping patients to know they care and encouraging continuity of care:

“It is obvious that this method of care provides the patient with the best outcomes. (#75, female, outer metropolitan)

There was a sense from some responses that PCC was a pervasive principle, that it was “emphasised by most clinicians” (#23, male, outer metropolitan), and another student said:

“Patient centred care has become a care ideology at all the clinical sites I attended so far this year (inner metropolitan hospital, general practice and specialist children’s hospital).” (#43, female, inner metropolitan)

By contrast, seven students had negative perceptions of PCC. They noted that they were encouraged to practice PCC, yet expressed quite cynical views about it. One student wrote that older patients don’t expect PCC, and another that it is the patient that achieves positive outcomes rather than medicine.

We identified three themes regarding how students were encouraged to practice PCC, which were through role modelling, direct teaching about PCC, and practising PCC. In each of these themes there were examples of positive and negative experiences (see Table 3). Positive role modelling of PCC was the most common influence, mentioned by 46 of the 99 respondents. Negative role modelling also featured, with students stating it had highlighted behaviours that they wanted to avoid. A few students cited the inclusion of patient centred principles in tutorials, case-based learning, and lectures. Finally, just ten students indicated active involvement in patient care, seven were in general practice settings and three did not mention the environment; and a few stated that being unable to practice PCC themselves was frustrating. Our analysis did not reveal any distinct patterns regarding PCC experiences according to clinical placement location or student gender.

**Table 3:** Influences encouraging patient-centred care

	Positive experiences	Negative experiences
Role modelling	“My Supervisor/mentor had a real emphasis on patient-centred care that will stay with me for the rest of my career.” (#127, male, inner metropolitan)	“The complete lack of patient centred care at the hospital has inspired me to do the opposite.” (#202, male, inner metropolitan)
	“GP especially was good for this (understanding PCC). My GP was great at collaborating with his patients regarding the best care for them.” (#83, female, inner metropolitan)	“Hospital is not patient centred” #131, female, rural)
Direct teaching	“Great teachers always make a point of this (patient-centred care) and it is important to always be aware of the whole person.” (#39, female, rural)	“We were told how patient centred care improves outcomes, but never how to actually practice it or given any opportunities to practice.” (#126, male, inner metropolitan)
Practising	“Especially during GP rotation. I’ve seen the same patient quite a few times and built a rapport which made me think about patient-centred care.” (#118, male, inner metropolitan)	“I have learned a lot about patients’ perceptions from interactions with them and often things that I end up having to tell the doctors because the doctor didn’t ask.” (#6, female, outer metropolitan)

### Other findings regarding role modelling

The 120 written responses regarding role modelling covered both *who* were role models, and *what behaviour* was observed. GPs were mentioned as positive role models by more students (*n* = 35) than any other discipline combined (*n* = 26). These were often compared with negative role modelling observed in other clinical settings. Paediatrics was the next most commonly experienced positive role modelling discipline. A handful of students mentioned women’s health, aged care, psychiatry or surgery. Specific disciplines were associated with negative role modelling such as surgery (*n* = 4) and obstetrics and gynaecology (*n* = 4), and GP (*n* = 10). Our analysis did not reveal any distinct differences in different clinical placement zones. The most commonly discussed positive role modelling behaviour related to various aspects of patient centred care, indicating that PCC and positive role modelling were integrally linked for students.

## Discussion

In addressing the main purpose of this study, we found that students’ self-perceived confidence did improve significantly after the two clinical years of the medical course, while their attitudes to patient centredness remained stable, albeit starting from a high baseline. The fact that they had very positive attitudes to patient centredness before their clinical placements may be partly explained by their exposure to principles of PCC in their first year simulated clinical environments, but also by their relative maturity as graduate level students. We did not find our predicted decline in attitudes to patient centredness that has been frequently described elsewhere [13]. This may reflect that the patient centredness scale we used was not responsive to small changes. However, if we trust that the scale was reliable enough to show large changes if they had existed, this seems to indicate a resistance to the negative role modelling that students reported. However, we also suggest that the graduate-entry nature of the student body may be providing some resistance to the biomedical influence, in that anti-PCC role modelling was regarded as negative by many of these students.

Students from all three clinical placement zones responded similarly on all measures at the beginning and the end of two years of clinical training. Our prediction that students’ patient centredness and confidence would be enhanced in the outer metropolitan and rural zones due to the increased exposure to community based medicine was not supported. It is possible that students’ minimal responsibility for patient care during their clinical placements prevented any real connection. Students connecting with and feeling responsible for individual patients was one of the most important factors in the success of the Harvard longitudinal clerkship program [19]. Further, we understood from the primary care community base program evaluation (not reported here) that students rarely had the opportunity to see the same patients over time during their weekly GP placements. So this lack of patient continuity failed to provide one of the known influences on patient centredness. Another important gap in our program was the minimal engagement with local communities to enable incorporation of their agenda regarding student placements in their area [22]. It is difficult, without this engagement, for students to develop meaningful connections outside of the clinical environment.

The qualitative analysis revealed that patient centeredness is encouraged by role modelling, direct teaching and opportunities to practise patient-centred care. One of the key influences on patient-centredness, regardless of location was the positive role modelling observed while at general practice placements. Students in all three zones had access to general practice experiences and all but the extended rural cohort had a five-week block placement in GP. The additional one day per week in general practice for two years for the outer metropolitan location students did not appear to additionally influence student confidence or patient centredness. We contend that this lack of additional influence likely relates to a lack of continuity of care with patients over time and inadequate opportunities for direct patient care experiences, both of which we hoped would be achieved in this setting as seen in the literature [15, 16]. Equally, continuity of clinical supervisor over the 2-year period did not appear to have any additional positive influence over having the GP supervisor for 5 full-time weeks. Additionally, ten students were critical of GPs with regard to role modelling for patient centredness, which was more than other disciplines. This reflects the high standards that they expected from their clinical supervisors, particularly GPs, and their expectations that GPs should be practicing patient centred medicine.

We suggest that the degree of similarity across the three zones is likely to arise from the centralisation of the medical curriculum, which was implemented using a distributed model of curriculum delivery with similar learning methods across all zones. Importantly, it also demonstrates that it is possible to deliver a complex medical curriculum in a variety of clinical locations including outer metropolitan and rural hospitals and in community settings that has been successful elsewhere in Australia [15].

Limitations are that students were all from a single institution and were all graduate-entry students, therefore limiting the transferability to other medical schools. The high level of patient centredness at baseline made it difficult to detect any improvements over time. The findings would also be enhanced if the confidence and patient centredness of the cohort could be followed to the end of their course and into their Internship. A further limitation is the use of self-report scales to measure attitudes, which has been critiqued recently [23]. Mead and Bower also criticised the tendency to use self-report scales to measure patient-centredness, suggesting that social desirability bias may influence responses [5]. This is particularly in settings where negotiation and empathy are valued characteristics amongst medical students. Further, the scales that we used had a potentially limited ability to detect changes, particularly in view of the relative low internal consistency of the patient-centredness scale. A new scale has recently been developed, the ‘self-efficacy in patient-centeredness questionnaire’, which has good validity and reliability and future studies may benefit from incorporating this measure [24]. Finally, there are many influences on confidence and attitudes towards patient centredness, and so it is difficult to suggest that any one factor such as more community-based placement time is influential.

A strength of this study was the relative similarity of all students at baseline, allowing comparison of any subsequent differences according to the clinical location. The study was a multi-site longitudinal study, contrasting with others in the field which are largely cross-sectional in nature, and involved a mixed method analyses of student experiences. The moderately high completion rate of both surveys also added robustness. The qualitative component of the study added a much more nuanced understanding of student attitudes to patient-centredness and the influential role of both positive and negative role-modelling in this regard. We suggest that qualitative approaches to understand attitudes to patient centredness such as interviewing students should be used in addition to validated surveys. Rees also advocates talking with students to understand their attitudes and motivations as well as observing their actual behaviours, as attitudes and clinical behaviours do not always correlate [25]. Finally, future studies need to ascertain whether positive attitudes towards patient centredness among medical students actually translate to patient centred clinical behaviour as doctors.

## Conclusion

Student confidence improved during the study period, and while attitudes to patient-centredness did not improve, they remained positive. The qualitative component of the study provided the most valuable insights into the influences that enabled retention of positive attitudes towards patient centredness. There were three substantive influences, namely role modelling, direct teaching and practising patient centredness. The most frequently discussed influence was role modelling, which included positive role modelling of patient centred behaviour amongst clinical staff, and also negative role modelling of non-patient centred care. Given that the majority of students regarded patient centredness as a pervasive ‘care ideology’, the negative role modelling only served to reinforce their commitment to patient centredness as a principle. Several students identified the ability to practise patient centred care as important but noted a lack of opportunity to do so. There is a need to increase the level of active student participation in patient care, with increasing levels of responsibility and opportunities to see the same patients over a long period of time. Creating sustained connection with patients is potentially important to consolidate students’ capacity to provide patient centred care in their future clinical practice.

## Abbreviations

CBME, community-based medical education; ERC, extended rural cohort; LIC, longitudinal integrated clerkships; PCC, patient-centred care; PCCB, primary care community base
